# Polyethylene Terephthalate-Based Composites with Recycled Flakes and Chemically Resistant Glass Fibres for Construction

**DOI:** 10.3390/polym17223087

**Published:** 2025-11-20

**Authors:** Krzysztof Adam Ostrowski, Paulina Romańska, Radosław Piech, Tomasz M. Majka, Adam Michalik, Dariusz Bednarowski, Zuzanna Zawadzka

**Affiliations:** 1 Faculty of Civil Engineering, Cracow University of Technology, ul. Warszawska 24, 31-155 Cracow, Poland; 2 Faculty of Materials Engineering and Physics, Cracow University of Technology, Al. Jana Pawła II 37, 31-864 Cracow, Poland; paulina.romanska@pk.edu.pl; 3 Interdisciplinary Center for Circular Economy, Cracow University of Technology, ul. Warszawska 24, 31-155 Cracow, Poland; radoslaw.piech@doktorant.pk.edu.pl (R.P.); tomasz.majka@pk.edu.pl (T.M.M.); 4 CUT Doctoral School, Faculty of Chemical Engineering and Technology, Cracow University of Technology, ul. Warszawska 24, 31-155 Cracow, Poland; 5 Department of Chemistry and Technology of Polymers, Faculty of Chemical Engineering and Technology, Cracow University of Technology, ul. Warszawska 24, 31-155 Cracow, Poland; zuzanna.zawadzka@student.pk.edu.pl; 6 ABB Corporate Technology Center, ul. Starowiślna 13A, 31-038 Cracow, Poland; adam.michalik@pl.abb.com (A.M.); dariusz.bednarowski@pl.abb.com (D.B.)

**Keywords:** polyethylene terephthalate, recycled PET, alkali-resistant fibres, fibre sizing, crystallisation behaviour, thermal degradation, impact performance, mechanical properties, composite microstructure

## Abstract

This study aims to evaluate the influence of glass fibre (GF) type and content on the thermal, mechanical, and morphological properties of polyethylene terephthalate (PET)-based composites containing post-consumer PET flakes, intended for mouldings exposed to cementitious environments (e.g., anchors). Two chemically resistant GFs were compared: alkali-resistant (AR) fibres with soft sizing (SGFs) and electrical-/chemical-resistant (ECR) fibres with hard sizing (HGFs). Composites with fibre contents of 10–60 wt.% were prepared, with detailed analysis focused on 30 to 50 wt.%—the range typical for practical applications. AR fibres experienced greater shortening during processing, and their actual fibre content was lower than the intended value. Differential scanning calorimetry (DSC) revealed enhanced crystallisation kinetics for SGF composites, with higher crystallinity of the injection-moulded samples and elevated crystallisation temperatures (206–208 °C for SGF vs. 196–197 °C for HGF). Thermogravimetric analysis (TGA) indicated that thermal stability was primarily governed by fibre content; both SGF and HGF composites showed improved resistance compared to neat PET. Mechanical tests demonstrated that both fibre types effectively reinforced the matrix: HGF composites exhibited a higher elastic modulus (20.6 GPa for HGF-50 vs. 18.3 GPa for SGF-50), whereas SGF composites exhibited roughly 10–20% higher tensile, flexural, and impact strength, despite slightly lower fibre content. SEM analysis confirmed more uniform fibre distribution and fewer voids in SGF composites. These results highlight the importance of GF selection for PET composites in alkaline environments, taking into account the properties of the sizing film former and balancing trade-offs between mechanical performance, thermal stability, processability, and economic factors.

## 1. Introduction

Polyethylene terephthalate (PET) is a widely used engineering thermoplastic recognised for its excellent mechanical properties, thermal resistance, and dimensional stability. It serves as a versatile matrix in composites across various sectors, including electrical and electronics, automotive, and machinery manufacturing. Driven by sustainability, recycled PET (rPET) is increasingly considered for non-packaging applications, although most post-consumer rPET is reclaimed for food and beverage packaging [1,2,3]. Industrial efforts, such as ENGEL’s two-stage process for the direct injection moulding of PET flakes (ENGEL, a manufacturer of injection moulding machines), indicate practical interest in broader applications [4].

In construction-related research, PET and rPET have predominantly been investigated as components of cementitious materials—particularly as aggregates and reinforcing fibres—as well as in the form of lightweight or insulating foams [5,6,7,8,9,10,11]. However, PET as a polymer matrix in glass fibre (GF)-reinforced composites remains underexplored, despite its favourable mechanical performance, creep and wear resistance, low water absorption, and dimensional stability, which make it a promising candidate for structural components exposed to moisture and concrete environments. Research on PET/GF and rPET/GF composites to date has primarily focused on conventional reinforcement with E-glass fibres. Early studies established that the incorporation of short GF into rPET significantly enhances stiffness and impact strength, making it a viable alternative to virgin engineering thermoplastics. Giraldi et al. reported a two- to threefold increase in these properties for rPET with 30 wt.% chopped GF [12], while Kračalík et al. demonstrated the crucial role of fibre–matrix adhesion in achieving high melt strength and mechanical integrity [13]. Mondadori et al. further showed that solid-state polymerised rPET combined with silane-treated GF offers superior strength and interfacial bonding [14]. Application-oriented research by Karthik et al. confirmed the potential of 30 wt.% GF-reinforced rPET for structural automotive components, meeting requirements for impact resistance, ageing, and durability while reducing CO_2_ emissions [15]. To mitigate brittleness, Monti et al. proposed reactive ethylene copolymers as effective impact modifiers, which improved toughness without compromising heat resistance [16]. More recently, Worku and Wubieneh demonstrated that hybrid rPET composites containing both GF and waste glass powder can achieve a favourable balance of strength, stiffness, and low water uptake, making them suitable for building applications [17]. In another approach, Vetterli et al. produced unidirectionally reinforced PET laminates by an in situ solid-state polymerisation route. Although this method represents an interesting and promising processing concept, the resulting mechanical performance remains lower than that typically achieved for injection-moulded short-fibre composites [18].

Despite their proven potential in structural applications, as evidenced by the studies cited above, the development of PET-based composites has never been directed towards construction uses involving exposure to alkaline or otherwise aggressive environments (e.g., anchors, fasteners, brackets). Conventional E-glass (electrical glass) was originally developed to provide favourable dielectric and mechanical properties; however, its composition—mainly based on SiO_2_, Al_2_O_3_, CaO, and B_2_O_3_—is not optimised for chemical durability, particularly in alkaline environments [19]. As a result, E-glass fibres are prone to degradation when exposed to high-pH pore solutions typical of cementitious materials, leading to embrittlement, loss of tensile strength, and deterioration of fibre–matrix adhesion [20]. To overcome these limitations, alkali-resistant (AR) glass fibres were developed by incorporating zirconium dioxide (ZrO_2_) into the glass composition. The zirconia-rich surface of AR-GF acts as a barrier, reducing hydroxyl ion diffusion and slowing the dissolution of the silica network. Paul et al. conducted an extensive durability assessment of coated AR- and E-glass textiles exposed to alkaline solutions and embedded in textile-reinforced concrete (TRC) composites [21]. Their results showed that AR glass textiles with a high zirconium content (15.6%) retained over 90% of their tensile strength after 56 days in simulated pore solution (pH 13.8), whereas E-glass textiles lost up to 56%. Microstructural analysis revealed significant pitting and coating degradation in E-glass yarns, while AR-GF remained largely unaffected. As noted by Peled, Bentur, and Mobasher, most TRC applications worldwide rely on AR-GFs because of their superior alkali resistance [22].

A more economical yet chemically durable alternative to AR glass is electrical-/chemical-resistant (ECR) glass. This type replaces boron and fluorine with more stable oxides—mainly SiO_2_ and Al_2_O_3_—and is designed to enhance chemical resistance without compromising mechanical or dielectric performance. ECR glass shows excellent stability in acidic and moderately alkaline environments and has become the industry standard for applications requiring long-term durability [23]. Benmokrane et al. evaluated the long-term performance of over 20 types of GFRP rebars, including those reinforced with E-glass, AR-glass, and ECR-glass [24]. Their accelerated ageing tests in NaOH, simulated pore solution, and moist concrete under sustained tensile loads showed that bars made with AR and ECR fibres exhibited significantly lower strength degradation than those with conventional E-glass. After 140 days in a simulated pore solution under a 30% sustained load, AR glass/vinyl ester bars retained over 89% of their tensile strength, whereas E-glass/vinyl ester bars lost up to 17%. SEM analysis confirmed more severe fibre damage and brittle fracture in E-glass specimens, while AR and ECR fibres exhibited broom-like failure modes indicative of more ductile behaviour. Further evidence was provided by Micelli et al., who studied GFRP reinforcement grids made with ECR GFs and epoxy–vinyl ester resin. After 30 days of immersion in Ca(OH)_2_ solution (pH 12.6), the specimens retained over 80% of their tensile strength with negligible loss in elastic modulus [25].

This study investigated PET-based composites incorporating PET flakes and reinforced with glass fibres exhibiting enhanced chemical resistance (ECR- and AR-type fibres), intended for potential structural applications. The approach was motivated by the ongoing need to develop cost-efficient and durable materials for construction, where both mechanical strength and long-term resistance to alkaline environments are crucial. The incorporation of PET flakes into the matrix addressed the growing industrial demand for recycled-content materials, while the replacement of conventional E-glass with chemically modified fibres provided a basis for further investigations into the ageing and durability of PET-based composites under alkaline exposure. The analysis assessed the thermal, mechanical, and structural properties of the materials. Two fibre loadings, 30 wt.% and 50 wt.%, were selected as the main variants, representing an optimal balance between processability and performance, and the maximisation of strength parameters. Preliminary tests were also carried out over a broader range of fibre contents (10–60 wt.%) to capture a wider perspective on composite behaviour. The two fibre types differed mainly in surface treatment and handling characteristics: ECR fibres, being stiffer and more cost-effective, were expected to enable easier compounding, whereas the more flexible AR fibres, though more expensive and less convenient to dose, were anticipated to provide a more effective overall performance.

## 2. Materials

PET (NEOPET^®^ 80) with an intrinsic viscosity of 0.80 ± 0.02 dl/g was purchased from NeoGroup (Vilnius, Lithuania). Typical mechanical properties are as follows: tensile strength ~55 MPa, tensile modulus ~3 GPa, and elongation at break ~45%. Recycled PET (RPET) flakes were supplied from Aloxe (Amsterdam, The Netherlands).

ECR glass fibres with standard (hard) silane-based sizing were purchased from Jar-mag Sp. z o. o. (Lublin, Poland). These chopped fibres had a nominal length of 3 mm and a nominal diameter of 10–15 µm. The alkali-resistant (AR) glass fibres were ARcoteX^®^ 5326, manufactured by Owens Corning^®^ (Toledo, OH, USA) using Cem-FIL^®^ AR glass chemistry. The AR fibres were treated with a silane-based soft sizing and had a nominal length of 3 mm and a nominal diameter of 14 µm. The selected ECR fibres represent a more economical alternative to the AR fibres, offering improved chemical resistance compared to standard E-glass at a significantly lower cost.

## 3. Sample Preparation

**Figure 1:**
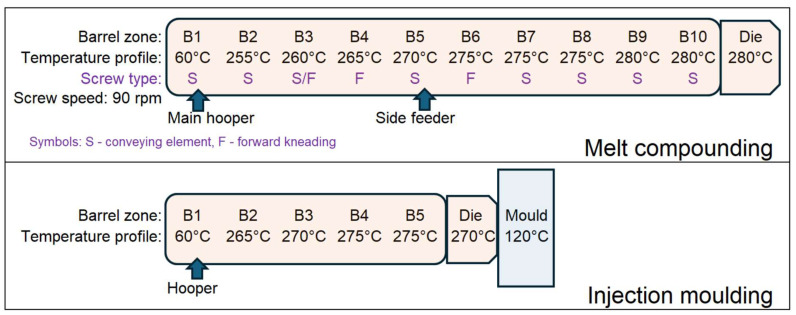
Scheme of the compounding and injection moulding process for HGF and SGF composites.

This work aimed to investigate the effect of the type of GF on the functional properties of PET/RPET composites. For this purpose, PET granulate and rPET flakes were pre-dried at 100 °C for 24 h (vacuum oven OV-11, Jeio Tech Co., Chalgrove, UK) to achieve a water content of ≤0.02%. The preparation process used a production line consisting of a Steer VF-20-Q feeder (Steer, Bangalore, India), a Steer Omega 20H co-rotating twin-screw extruder (D = 20 mm, L/D = 44), a cooling tank, and a Maag SGS 25-E4 pelletizer (Maag Automatik GmbH, Großostheim, Germany). The processing parameters are schematically illustrated in Figure 1. Using the melt compounding process, composites were produced containing either softer AR glass fibres (SGFs) or harder ECR glass fibres (HGFs), each with 5 wt.% recycled PET flakes and a total of 10, 20, 30, 40, 50, or 60 wt.% of either AR or ECR fibres. The low mass fraction of rPET flakes was justified by their limited external availability, as most flakes are retained by producers for regranulation and primarily used in packaging.

Standard samples for mechanical tests, both dumb-bell-shaped specimens and rectangular bars, were prepared with a cross-section of 4 × 10 mm using a KM 30 125C injection moulding machine (Krauss Maffei, Munich, Germany). The barrel temperature profile was set in the range of 265–275 °C, and the mould temperature was maintained at 120 °C.

In the text, tables, and figures, the following sample designations are used for clarity. The reference material (**REF**) corresponds to the PET matrix containing 5 wt.% recycled PET flakes. The remaining samples consist of the same PET matrix with 5 wt.% PET flakes, further reinforced with GFs, differing in chemical composition and surface sizing. The two types of fibres were designated as follows: **HGF**—ECR glass fibres with hard (standard) sizing, and **SGF**—AR glass fibres with soft sizing. The numerical value in the designation (e.g., 10-HGF, 30-SGF) indicates the nominal fibre content in the composite, expressed as the weight percentage of fibres in the total material. For example, the designation 30-HGF refers to a composite of PET + 5 wt.% PET flakes reinforced with 30 wt.% ECR GF with hard sizing, while 50-SGF refers to a composite of PET + 5 wt.% PET flakes reinforced with 50 wt.% AR GF with soft sizing.

## 4. Methods

To determine the fibre mass fraction and fibre length distribution via the burn-off test, the samples were incinerated in a muffle furnace at 600 °C for a total duration of three hours per sample. The fibre mass fraction was determined in accordance with ISO 3451-1:2019, Method A (rapid ashing) [26]. Following the removal of the matrix, the fibre length was measured in compliance with ISO 22314:2023, using a Keyence VHX-7000 optical microscope (Keyence International, Mechelen, Belgium) [27]. The fibres were placed on a microscope slide immersed in water containing a Tergitol 15s9 surfactant and subsequently dispersed using an ultrasonic cleaner. The dataset comprising measured fibre lengths was analysed using MATLAB R2025a (The MathWorks, Natick, MA, USA) to determine the statistical fibre length distribution (FLD) and to extract representative parameters describing the fragmentation behaviour of fibres within the composite. Initially, the raw data (individual fibre lengths) were imported into MATLAB as a vector. Histograms of the fibre length distribution were then plotted using the *histogram* function. The two-parameter Weibull distribution was fitted to the data. The parameters of the Weibull distribution—the shape and scale parameters—were estimated using maximum likelihood estimation implemented in MATLAB’s *wblfit* function. The resulting PDF of the Weibull distribution was calculated using the *wblpdf* function and superimposed on the histogram to assess the goodness of fit visually. Additionally, two standard scalar metrics were computed directly from the measured dataset to summarise the FLD: number-average fibre length (1) and weight-average fibre length (2):

(1) $${\bar{L}}_{n}=\frac{1}{N}\sum_{i=1}^{N}L_{i}$$

(2) $${\bar{L}}_{w}=\frac{\sum_{i=1}^{N}{L_{i}}^{2}}{\sum_{i=1}^{N}L_{i}}$$

where ${\bar{L}}_{i}$ is the length of the *i*-th fibre, and *N* is the total number of measured fibres.

Thermogravimetric analysis (TGA) was performed on the NETZSCH TG 209F1 Libra apparatus (Netzsch, Selb, Germany). The test was carried out in an oxidising atmosphere under the following conditions: temperature range from 30 to 600 °C; heating rate—10 °C/min. Each sample was tested in an open measuring cell made of Al_2_O_3_, with a sample weight of approximately 5 mg.

The polymer composites were examined for thermal transitions using differential scanning calorimetry (DSC) on a Mettler Toledo DSC 823e (Mettler Toledo, Warsaw, Poland) apparatus. The measurements were performed in a nitrogen atmosphere according to the following temperature programme: heating from 25 to 300 °C at a rate of 10 °C/min, cooling from 300 to 25 °C at a rate of 10 °C/min, and a second heating from 25 to 300 °C at a rate of 10 °C/min. Characteristic transition temperatures and enthalpies were determined from the DSC curves. The degree of crystallinity is calculated as follows:

(3) $$\chi =\frac{∆H_{m}-∆H_{cc}}{(1-\omega )∆H_{m}^{0}}\cdot 100\%$$

where $∆H_{m}$, $∆H_{cc}$ are the experimental melting enthalpy and cold crystallisation enthalpy, respectively, $∆H_{m}^{0}$= 120 J/g is the literature data for the equilibrium melting enthalpy of 100% crystalline PET [14], and ω is the actual fibre weight fraction measured with the burn-off method.

The static tensile properties of the materials were determined using a Shimadzu AGS-X testing machine (Shimadzu, Wrocław, Poland) in accordance with the ISO 527-1:2019 standard, at a crosshead speed of 1 mm/min for modulus determination and 5 mm/min for the remaining measurements [28].

The static flexural strength and maximum strain of the obtained composite materials were tested using a Zwick Z005 TH Allround-Line (ZwickRoell Sp. z o. o. Sp. k., Wrocław, Poland) universal testing machine, in accordance with ISO 178:2019, at a deformation rate of 2 mm/min [29].

The Charpy impact test was performed using the Zwick HIT5.5P machine (ZwickRoell Sp. z o. o. Sp. k., Wroclaw, Poland) by EN-ISO 179-1:2023 [30]. The test was performed using a rigid impact hammer with an impact energy of 2 J. The notched and unnotched impact strengths were calculated.

The mechanical tests were conducted on specimens conditioned at 23 ± 2 °C and 50 ± 5% relative humidity, in accordance with standard laboratory practice. Five specimens were tested for each data point, while in rare cases, four valid results were used for statistical evaluation due to specimen imperfections.

## 5. Results and Discussion

### 5.1. Ash Content and FLD

The burn-off tests provide insight into the actual filler content (Table 1). In composites with soft-sized GFs (SGFs), the measured fibre mass fraction was roughly five percentage points lower than the nominal target values (30 and 50 wt.%), indicating inefficient incorporation during processing. In contrast, composites reinforced with hard-sized GFs (HGFs) showed fibre contents very close to the intended values, with only a minor deviation of about 1% in the 30 wt.% HGF system. These differences may reflect variations in dosing behaviour resulting from the distinct surface treatment of the fibres (e.g., the protective effect of rigid sizing) and the generally higher bulk density of HGFs, which enables more accurate dosing.

**Figure 2:**
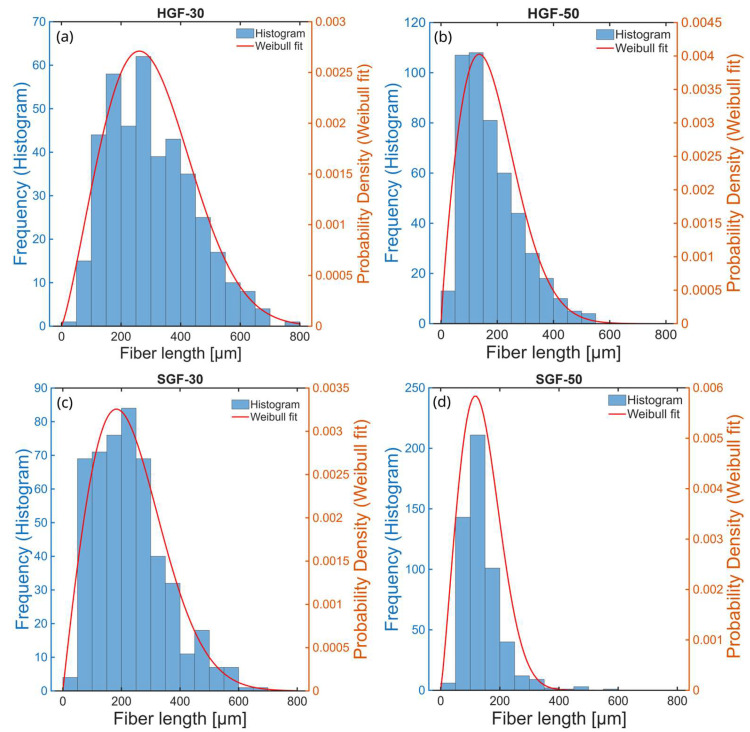
Fibre length distribution depending on the type and amount of GFs: (**a**) 30 wt.% ECR fibres, (**b**) 50 wt.% ECR fibres, (**c**) 30 wt.% AR fibres, (**d**) 50 wt.% AR fibres.

**Figure 3:**
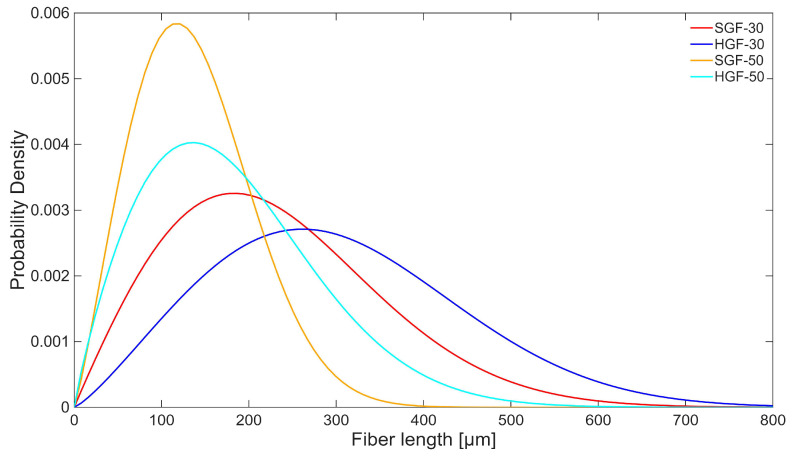
Weibull-fitted fibre length distributions for the tested composites.

Figure 2 presents the fibre length histograms for the tested composites, which follow a Weibull distribution. The comparison of fibre length distributions, based on fitted Weibull probability density functions (PDFs, Figure 3), revealed a clear trend of increasing fibre fragmentation with higher fibre volume fraction in the composites. This effect, commonly observed in fibre-reinforced materials, resulted from the increased degree of fibre breakage during processing at higher fibre loadings. More importantly, a distinct difference between HGF and SGF fibres was evident. For fibres with soft sizing (SGFs), the shift toward shorter fibre lengths was more pronounced, indicating that these fibres were more susceptible to breakage despite their slightly lower actual content in the composite. This observation was further confirmed by the calculated number- and weight-average fibre lengths (Table 1). The combined analysis of fibre length statistics and filler content showed that the greater fragmentation and lower effective content of SGFs may have reduced reinforcement efficiency, whereas HGF composites retained both fibre integrity and the expected volumetric contribution.

### 5.2. DSC Results

Differential scanning calorimetry revealed distinct differences between the reference PET and the GF-reinforced composites, as well as between samples containing fibres with hard (HGF) and soft (SGF) sizing. The thermal behaviour was affected both by the fibre surface treatment and by the fibre content. This is clearly demonstrated in Table 2 by the differences in the characteristic transition temperatures (particularly the crystallisation temperature) and the degrees of crystallinity of the materials.

During the first heating, all composites exhibited a cold crystallisation peak, indicating incomplete crystallisation of the matrix during cooling in the mould. The addition of GFs reduced the melting enthalpy (Δ*H*_m1H_) and, for hard-sized fibres, also decreased the degree of crystallinity (*χ*_1H_) compared to the reference. In contrast, SGF composites maintained crystallinity values close to that of unfilled PET, indicating that the soft-sized fibres did not significantly hinder chain mobility during cooling. Increasing the GF content from 30 wt.% to 50 wt.% further reduced Δ*H*_m1H_ and enthalpy of cold crystallisation (Δ*H*_cc_) for HGF composites, reflecting a stronger restriction of polymer chain mobility at higher fibre loadings.

During the cooling stage, all composites crystallised at a temperature above the reference (*T*_c_ = 195 °C), with SGF composites exhibiting the highest *T*_c_ values (~206–208 °C), indicating a more pronounced nucleating effect of the soft-sized fibres.

**Figure 4:**
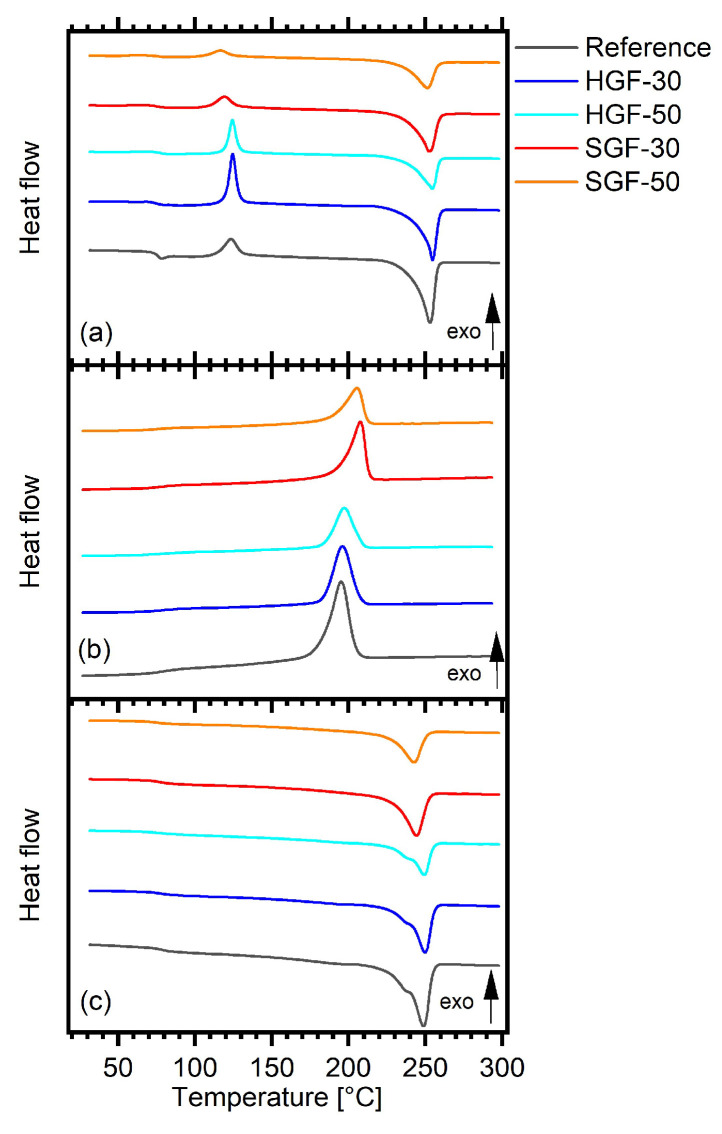
DSC curves for the first heating (**a**), cooling (**b**), and second heating (**c**).

During the second heating, no cold crystallisation was detected for any of the samples, indicating that the differences in their previous thermal histories had been erased after the first complete heating–cooling cycle. The absence of this transition suggests that the amorphous regions capable of further ordering had already crystallised during the controlled cooling step of the DSC scan. As a consequence, both the reference PET and the HGF composites exhibited a higher degree of crystallinity compared to the first heating, whereas the SGF composites showed nearly unchanged χ_2H_ values. This suggests that in the case of SGFs, the potential for further ordering of the molecular chains had already been largely exploited during processing. Moreover, during the second heating, the melting endotherms of the reference and HGF composites exhibit a low-temperature shoulder preceding the main melting peak—the DSC curves are shown in Figure 4. This feature is typically associated with a reorganisation of less stable crystallites during heating, as described by Frenzel et al. [31] for PET/GF systems with various sizing types. In contrast, this effect was not observed in SGF composites, suggesting the formation of a more homogeneous, fine-grained crystalline structure.

The measured glass transition temperatures (*T*_g_) of the composites and REF fell within a narrow range of 74–78 °C, indicating only minor differences among the materials. However, slightly the higher *T*_g_ of HGF-50 during the first heating and the somewhat lower *T*_g_ of SGF-50 during the second heating correspond well with the discussed influence of fibre type and sizing on polymer chain mobility and morphology.

Overall, the results suggest that SGFs, compared to HGFs and REF, have a pronounced nucleating effect, leading to faster crystallisation and a higher degree of crystallinity in injection-moulded parts, likely producing relatively small and homogeneous crystals. By comparison, the nucleating effect in HGFs is noticeably weaker, resulting in a crystalline structure that may be less uniform.

### 5.3. TGA Results

**Figure 5:**
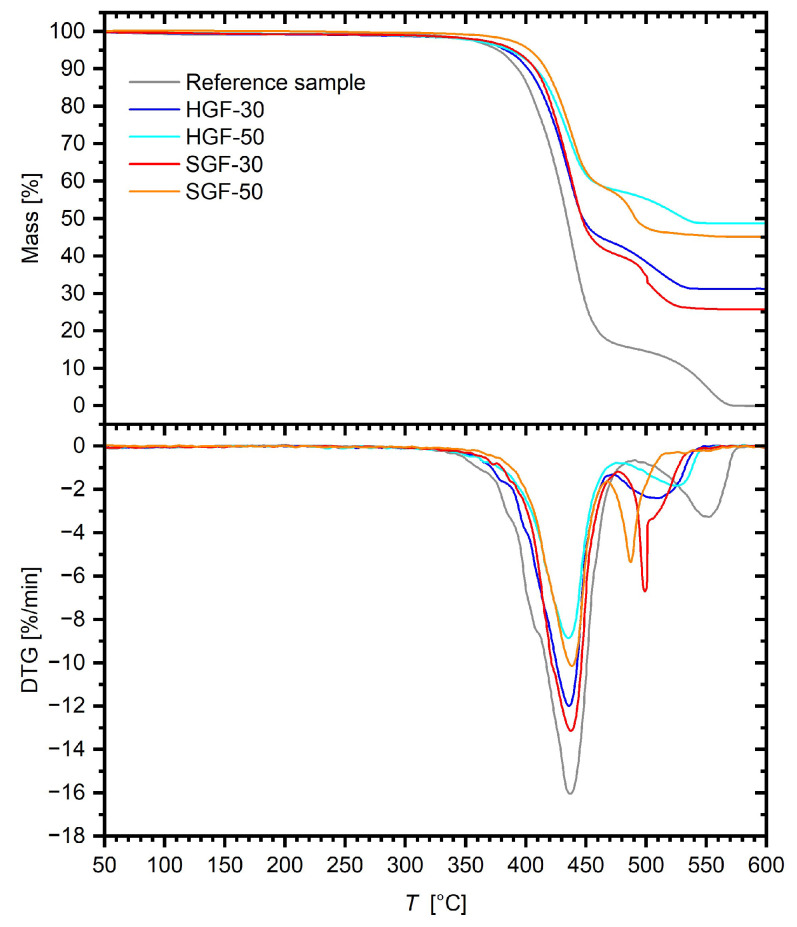
TG and DTG test results for the tested materials.

Data illustrating the mass loss of the samples are presented in Figure 5, while the most important values obtained from the graphs are summarised in Table 3. The parameters *T*_5%_, *T*_10%_, *T*_20%_, and *T*_50%_ correspond to the temperature at which the samples’ mass decreased by 5%, 10%, 20%, and 50%, respectively. The analysis was conducted in an oxidising atmosphere, which allows for the assessment of the material’s stability under actual use conditions, such as exposure to air at elevated temperatures.

The residual mass at the end of the process for HGF and SGF composites was generally consistent with the values obtained from matrix burn-off measurements (Table 1), confirming that the dosing of hard-sized fibres was more accurate, whereas the actual content of soft-sized fibres was slightly lower than intended, particularly for SGF-50.

The reference sample demonstrated a two-stage thermal decomposition. The first stage, occurring up to approximately 380 °C, was primarily associated with the removal of moisture and volatile substances, as well as the initiation of polymer chain degradation. The second stage, observed above this temperature, corresponded to the actual decomposition of the matrix and depolymerization of the main polymer segments. For the reference sample, the *T*_5%_ was 377 °C, and the *T*_10%_ was 393 °C. As the temperature increased to 408 °C (*T*_20%_) and 434 °C (*T*_50%_), the degradation process intensified, reaching a maximum mass loss rate (*T*_MAX_ = 437 °C). These values are typical for PET and indicate that the main degradation occurs within a narrow temperature range, suggesting a homogeneous degradation mechanism. For samples containing GFs, a notable increase in thermal resistance was observed across the entire temperature range. With increasing GF content, the *T*_5%_, *T*_10%_, *T*_20%_, and *T*_50%_ values shifted toward higher temperatures, confirming the stabilising effect of the fibres. However, the impact of fibre type varied depending on the temperature range. SGF composites showed slightly better stability in the initial stages of degradation, while HGF composites outperformed at higher temperatures. In HGF samples, the first stage of degradation began slightly earlier than in SGF ones, which can be attributed to the presence of a larger amount of fibre surface with residual sizing susceptible to oxidation. However, in the second stage, the degradation process of HGF composites was significantly slower. This means that hard-sized fibres, which exhibited a somewhat higher mass fraction and greater average fibre length (Table 1) than SGF composites, create a more effective barrier, limiting oxygen diffusion and heat transfer into the material, thereby slowing down oxidation and further depolymerization of the matrix. It is worth noting that the fibre content exerted a more decisive influence on thermal stability than the fibre-sizing chemistry. This is exemplified by the increasing differences in *T*_50%_ values between SGF and HGF composites with rising GF content—for composites with 30 wt.% GF, the difference was only 1 °C, whereas for 50 wt.% GF, it reached 41 °C. A significant effect of filler content has also been observed in other studies, including Sathishkumar et al. [32].

The TGA results demonstrate that the addition of GFs significantly improved the thermal stability of the material, and the effectiveness of this reinforcement depends on both the amount and geometry of the fibres. AR fibres with soft sizing provided stabilisation at lower temperatures, whereas ECR hard-sized fibres played a dominant role at higher temperatures, offering more effective protection against oxidative degradation. It should be noted that the mass fraction of the sizing itself in the composites is negligible and does not produce a discernible TGA signal. Based on literature reports, thermal degradation of the sizing in air occurs over a temperature range of 200–500 °C, being particularly pronounced between 300 and 400 °C [33,34]. Although the thermal–oxidative behaviour of PET-based composites is mainly determined by the polymer matrix, the presence of appropriately sized GFs, as suggested by other reports in the literature, may improve thermal stability and extend the material’s effective temperature–time limits [34].

### 5.4. Mechanical Test Results

**Figure 6:**
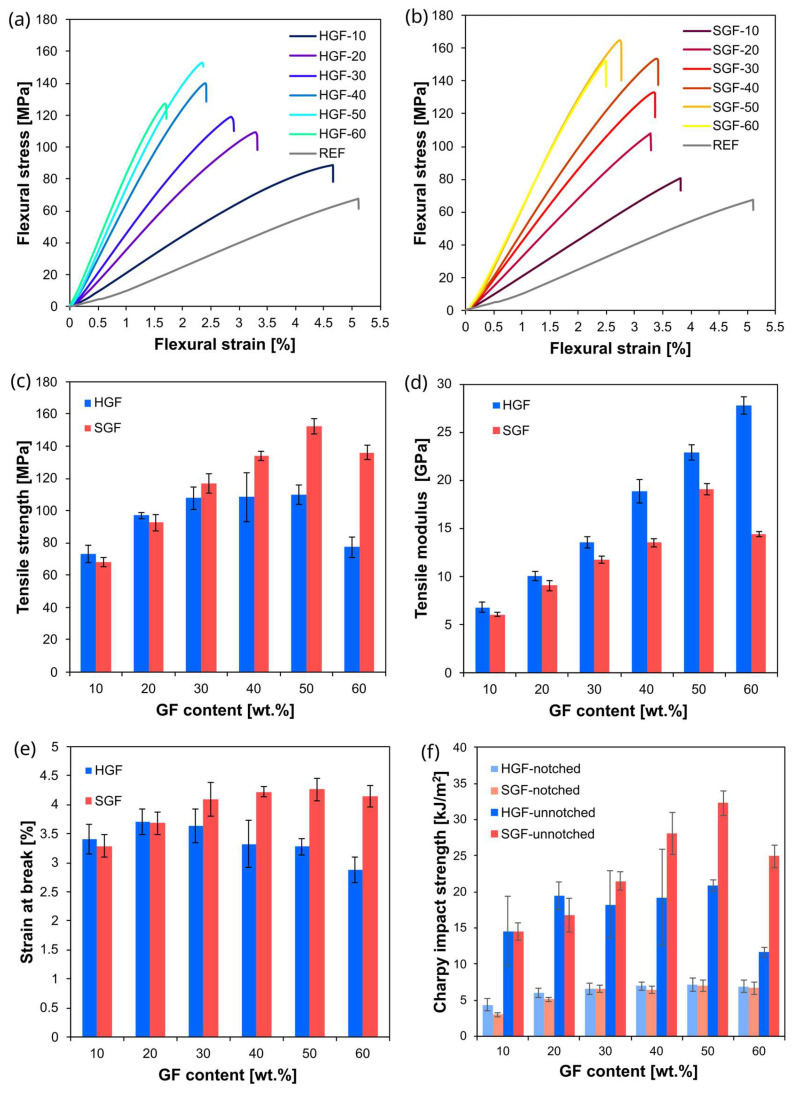
Summary of the mechanical test results: (**a**,**b**) representative bending curves for REF, HGFs, and SGFs; (**c**–**e**) summary of the main tensile test results; (**f**) Charpy impact strength of the tested materials with and without a notch.

Composites with two typical GF loadings (30 wt.% and 50 wt.%) were selected for the majority of analyses, as they represent fibre contents commonly used in practical applications. To more accurately capture trends in material behaviour, mechanical tests were also performed across a broader range of fibre contents. The results of these tests—including tensile and flexural properties and the impact strength of both notched and un-notched specimens (to evaluate impact resistance and notch sensitivity)—are summarised in Table 4. To enhance the clarity of the results, selected data—including bending curves as well as tensile and impact parameters—are additionally presented in graphical form in Figure 6.

The tensile and flexural test results showed the expected trends for fibre-reinforced composites: both the elastic modulus and the strength increased with increasing fibre content, while the materials remained distinctly brittle, exhibiting low elongation at break (2–5%). Notably, the reference material itself, composed of 95 wt.% PET and 5 wt.% recycled PET flakes, exhibited brittle behaviour. The elongation at break decreased markedly from ~50% for virgin PET to ~5% for the tested matrix, despite extrusion compounding. When GFs were incorporated, both the quality of the mouldings and the impact resistance improved, particularly for SGF composites. Thus, although the addition of 5 wt.% PET flakes reduced deformability in the fibre-free system, this adverse effect was mitigated in the fibre-reinforced materials. They exhibited the expected, typical properties of PET-based materials.

The increase in elastic modulus with fibre content was more pronounced in HGF composites, particularly at 60 wt.% GF, where the modulus reached ~27 GPa compared to ~14 GPa for SGF. In SGF composites, the modulus increased steadily up to 50 wt.% GF but declined at 60 wt.% GF due to excessive fibre fragmentation. Notably, for both fibre types, a fibre content of 60 wt.% no longer provided effective reinforcement in terms of strength, as both tensile and flexural strengths decreased compared to composites containing 50 wt.% fibres. This behaviour is typical of thermoplastic composites containing very high contents of short, dispersed fibres, where processing difficulties, fibre breakage, and poor impregnation outweigh the potential reinforcement effect.

In contrast, the tensile strength increased more markedly for SGF composites, reaching a maximum of ~154 MPa at 50 wt.%, compared to ~125 MPa for the HGF ones at the same fibre content. Comparable trends were observed for flexural strength. This difference in reinforcement efficiency between HGFs and SGFs is particularly notable since the fibres in the SGF composites were both shorter than in the HGF composites and present at a slightly lower actual mass fraction. This finding highlights strong interfacial bonding, which reduces the critical fibre length, enabling the shorter AR fibres to contribute effectively to stress transfer up to 50 wt.% fibre content.

Impact strength measurements revealed further differences related to the factors that influenced the properties observed in the static tests. In notched specimens, both types of fibre-reinforced composites demonstrated higher impact resistance than the REF, with only minor differences between SGFs and HGFs at comparable fibre contents. However, in unnotched specimens, the differences became more evident, especially at higher fibre content, where the SGF composites outperformed the HGF composites. At 50 wt.% GF, the unnotched impact strength of the SGF composite exceeded that of the HGF ones by more than 11 kJ/m^2^.

The observed advantage of SGFs over HGFs in tensile, flexural, and notched impact strength can be primarily attributed to the overall structural coherence of the material and strong matrix–fibre interactions (see Section 5.4 for further discussion). This is likely complemented by a favourable, homogeneous crystalline structure, as suggested by DSC results.

### 5.5. Microscopic Analysis

**Figure 7:**
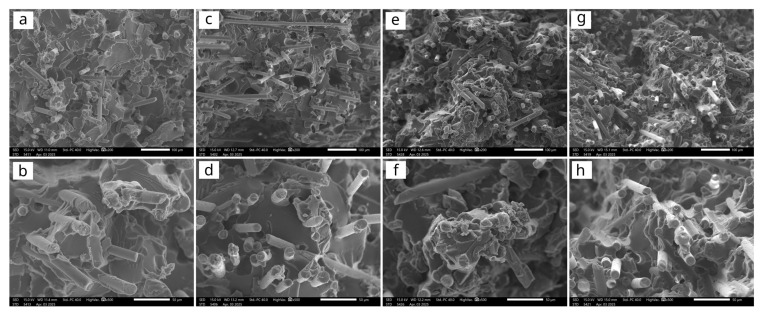
SEM images of tensile fracture surfaces of SGF-30 (**a**,**b**), HGF-30 (**c**,**d**), SGF-50 (**e**,**f**), and HGF-50 (**g**,**h**). The top row shows images taken at 200× magnification, while the bottom row displays images at 500× magnification.

Figure 7 shows representative SEM images of the fracture surfaces of SGF and HGF composites with 30% and 50% fibre content. SEM analysis of the fracture surfaces obtained from tensile tests of both SGF and HGF composites revealed a predominantly brittle fracture character in all cases. The fibre lengths pulled out from the matrix were comparable between the two fibre types, indicating a similar critical fibre length in both systems. The matrix exhibited sufficient adhesion to the fibres in both composite types, as evidenced by the absence of gaps at the fibre–matrix interfaces and the relatively short fibre pull-out lengths observed.

However, while the overall distribution of fibres appeared relatively uniform in both types of composites, the HGF samples exhibited somewhat poorer fibre dispersion (isolated clusters), which may have adversely affected the reinforcement efficiency. The presence of voids and discontinuities within the fracture surfaces of the HGF composites is also noticeable (most clearly seen in Figure 7c), despite processing under the same conditions as the SGF composites. These microstructural defects indicate that fibres with hard sizing, being stiff and assembled in relatively thick strands, may hinder proper fibre impregnation with the matrix, limit fibre dispersion during compounding, and adversely affect the mould filling during injection moulding, compared to SGF. This microstructural inhomogenisation is likely the main factor contributing to the reduced tensile and flexural strength, as well as the increased brittleness, observed in the HGF composites. The elastic modulus is less sensitive to such defects, as it depends more strongly on the fibre length, which is primarily affected by fibre fragmentation during processing.

These observations suggest that the finer dispersion of SGF fibres, together with the absence of voids that would otherwise act as stress concentrators, contributes more effectively to energy dissipation under dynamic loading, particularly at higher reinforcement levels in unnotched specimens. Softening or increasing the flexibility of the film former in the sizing formulation can produce softer and more compliant GF strands, which facilitates their dispersion and wetting by the molten polymer during compounding and moulding [35].

## 6. Conclusions

Soft-sized AR-GFs and hard-sized ECR-GFs used in PET-based composites intended for alkaline environments both effectively reinforced and toughened the brittle PET/rPET matrix, each offering distinct advantages while presenting certain limitations. AR fibres were more challenging to dose, resulting in slightly lower actual fibre content and higher susceptibility to fragmentation. Nevertheless, they enabled particularly high tensile and flexural strength, as well as improved unnotched impact resistance, which can be attributed to homogeneous fibre dispersion, absence of structural defects, and likely a fine-grained, homogeneous crystalline structure. DSC revealed faster crystallisation kinetics and a pronounced nucleating effect for AR-GF composites, and TGA indicated a slightly delayed onset of thermal degradation compared to hard-sized ECR fibre composites. In contrast, the introduction of ECR fibres was economically more advantageous, providing a marked increase in elastic modulus and slower thermal degradation in air at higher temperatures. However, despite more precise dosing and limited fibre fragmentation during processing, ECR composites exhibited somewhat lower tensile, flexural, and impact strength, likely due to structural discontinuities caused by the rigid surface sizing, which may hinder matrix impregnation. Despite these drawbacks, the overall performance of both types of composites was satisfactory, making them suitable for further ageing studies in alkaline environments. This study demonstrated that the tested composites have strong potential for civil engineering applications, such as injection-moulded anchors for cementitious or rock substrates, load-bearing components, and other industrial uses. The addition of waste-derived rPET further reduces the carbon footprint and supports the move toward sustainable, resource-efficient construction.

## Figures and Tables

**Table 1:** Characterisation of fibre length and weight fraction.

Material	Number-Averaged Fibre Length [µm]	Weight-Averaged Fibre Length [µm]	GF Weight Fraction [%wt.]
HGF-30	301	370	28.9
HGF-50	231	298	50
SGF-30	180	239	26.1
SGF-50	137	169	44.9

**Table 2:** Summary of the key DSC data for the tested materials. Indices: 1H—first heating cycle; 2H—second heating cycle; *T_g_*—glass transition temperature; *T*_m_—melting temperature; *T*_cc_—cold crystallisation temperature; *T*_c_—crystallisation temperature; Δ*H*_cc_—enthalpy of cold crystallisation; Δ*H*_m_—enthalpy of melting; Δ*H*_c_—enthalpy of crystallisation; χ—degree of crystallinity (%).

Material	*T*_g1H_ [°C]	*T*_cc1H_ [°C]	*T*_g2H_ [°C]	Δ*H_cc_* [J/g]	*T*_m1H_ [°C]	Δ*H*_m1H_ [J/g]	*χ*_1H_ [%]	*T*_c_ [°C]	Δ*H*_c_ [J/g]	*T*_m2H_ [°C]	Δ*H*_m2H_ [J/g]	*χ*_2H_ [%]
REF	74.46	123.63	78.53	10.10	253.16	45.83	29.8	195.24	41.86	248.90	41.71	34.8
HGF-30	75.06	124.71	76.83	18.43	254.70	37.52	22.4	196.03	31.26	249.93	29.86	35.0
HGF-50	77.44	124.51	76.23	12.10	254.63	26.31	23.7	197.31	22.12	249.33	21.30	35.5
SGF-30	75.81	119.46	76.67	7.00	252.58	33.15	29.5	207.88	27.73	244.44	26.22	29.6
SGF-50	75.88	116.79	74.44	3.83	251.45	23.02	29.0	205.66	19.02	242.81	20.75	31.4

**Table 3:** Key data from the TG and DTG curves for the tested materials.

Material	*T*_5%_ [°C]	*T*_10%_ [°C]	*T*_20%_ [°C]	*T*_50%_ [°C]	*T*_MAX_ [°C]	Remaining Mass [%]
REF	377	393	408	434	437	0.00
HGF_30	383	401	418	448	436	31.18
HGF_50	387	409	426	532	435	48.67
SGF_30	390	408	422	447	438	25.77
SGF_50	403	417	430	491	438	45.03

**Table 4:** Summary of mechanical test results: tensile modulus (*E*_t_), tensile strength (σ_tM_), tensile strain at break (ε_tM_), flexural strength (σ_fM_), flexural strain at break (*ε*_fB_—equal to flexural strain at maximum force), notched (*a*_cN_), and unnotched (*a*_cU_) Charpy impact strength.

Material	*E*_t_ [GPa]	σ_tM_ [MPa]	ε_tM_ [%]	σ_fM_ [MPa]	ε_fB_ [%]	*a*_cN_ [kJ/m^2^]	*a*_cU_ [°C]
REF	2.980 ± 0.511	56.2 ± 1.8	4.9 ± 0.3	61.8 ± 5.0	4.4 ± 0.6	3.65 ± 1.05	38.3 ± 7.86
HGF-10	5.303 ± 0.392	78.1 ± 1.3	4.4 ± 0.1	92.8 ± 3.8	5.0 ± 0.7	4.35 ± 0.81	14.52 ± 4.84
HGF-20	9.909 ± 0.432	107.5 ± 4.7	4.2 ± 0.1	112.35 ± 3.1	3.4 ± 0.1	5.96 ± 0.66	19.52 ± 1.91
HGF-30	12.522 ± 0.481	109.4 ± 4.0	3.9 ± 0.2	118.0 ± 3.0	2.8 ± 0.1	6.55 ± 0.83	18.23 ± 4.63
HGF-40	17.813 ± 0.253	121.2 ± 4.5	3.7 ± 0.2	138.8 ± 6.0	2.5 ± 0.1	6.94 ± 0.58	19.19 ± 6.64
HGF-50	20.609 ± 0.040	124.9 ± 9.8	3.6 ± 0.2	144.5 ± 7.8	2.2 ± 0.2	7.11 ± 0.90	20.82 ± 0.81
HGF-60	26.790 ± 1.627	108.5 ± 14.7	2.9 ± 0.3	120.8 ± 5.8	1.6 ± 0.1	6.90 ± 0.90	11.68 ± 0.63
SGF-10	5.059 ± 0.119	64.6 ± 1.9	3.4 ± 0.1	70.9 ± 7.8	3.3 ± 0.5	2.97 ± 0.24	14.49 ± 1.22
SGF-20	8.376 ± 0.124	103.2 ± 0.9	4.2 ± 0.2	105.5 ± 6.2	3.4 ± 0.1	5.10 ± 0.28	16.71 ± 2.32
SGF-30	11.311 ± 0.301	124.4 ± 0.6	4.8 ± 0.2	131.8 ± 9.6	3.5 ± 0.2	6.59 ± 0.53	21.48 ± 1.29
SGF-40	12.911 ± 0.319	132.7 ± 4.2	4.7 ± 0.3	151.5 ± 3.2	3.3 ± 0.1	6.48 ± 0.50	28.06 ± 2.91
SGF-50	18.304 ± 0.126	154.2 ± 6.0	4.4 ± 0.2	159.1 ± 4.5	2.7 ± 0.3	7.00 ± 0.80	32.25 ± 1.70
SGF-60	13.829 ± 0.379	137.6 ± 2.1	4.6 ± 0.2	158.2 ± 11.0	2.8 ± 0.3	6.65 ± 0.81	24.89 ± 1.53

## Data Availability

The original contributions presented in this study are included in the article. Further inquiries can be directed to the corresponding authors.
